# Authors' Reply to Comments from Dr. Guan

**DOI:** 10.1155/2009/215935

**Published:** 2009-12-24

**Authors:** Ken-ichiro Inoue, Hirohisa Takano

**Affiliations:** Environmental Health Sciences Division, National Institute for Environmental Studies, 16-2 Onogawa, Tsukuba 305-8506, Japan

*To the Editor*. We thank Dr. Guan for his interest in our recent article [1]. He has raised some comments regarding possible potential of MT and zinc as therapeutic tools for inflammatory diseases. As he mentions, simply inducing and/or enhancing metallothionein (MT) is harmful, particularly in physiological condition. We want to emphasize that for severe (lethal) inflammatory conditions such as systemic inflammatory response syndrome (SIRS) including acute lung injury and fulminant hepatitis in humans, in which proper therapeutic strategy has not been established yet, our findings using MT knockout mice may open doors to the alternative therapeutic target. Of course, there seems to be a large volume of problems and issues to be overwhelmed/addressed as Guan points out; thus, considering this, we had concluded with toning down that “…implicating MT-induction/enhancement and/or zinc supplementation to induce/enhance MT as possible therapeutic options for inflammatory diseases, although additional research is needed to conclude its clinical utility.” 

As for zinc supplementation, it should be taken granted that too much zinc is harmful to health in physiological state, as Guan insists. Experimentally, however, zinc supplementation shows therapeutic and preventional effects against several pathologies such as diabetic nephropathy [2] parasite infection [3], *H. pylori*-related gastritis [4], as well as sepsis [5]. In addition, it was reported that zinc deficiency increases organ damage and mortality of sepsis *in vivo* [6], suggesting that zinc supplementation can protect from the pathophysiology in the disease. Therefore, it is attractive to speculate that zinc supplementation could be alternative therapeutic option against refractory diseases including severe inflammatory ones such as SIRS, if one carefully monitors zinc concentration.

The role of MT in carcinogenesis remains controversial as Guan describes. MT (−/−) mice are reportedly susceptible to metal- and chemical-induced carcinogenesis [7, 8]. Carcinogenesis involves much mechanistic pathways dependent on stimuli and/or affected cells/organs; thus, we understand that additional *in vivo* tests and careful clinical trials are required to employ the molecule (MT) to therapeutic options in view of tumorgenesis. Unfortunately, in addition, we had failed to obtain recombinant mouse MT in our previous studies, to validate pharmaceutical rescue for MT (−/−) mice. We want to conduct the experiments in the future.

Finally, we hope we will continue basic research regarding MT in pathophysiological conditions paying attention to the points Dr. Guan states in his comments.

*Ken-ichiro Inoue* *Ken-ichiro Inoue* *Hirohisa Takano* *Hirohisa Takano*
